# Mucoprotectants and gut barrier: mechanisms of action and clinical applications in IBS. Is there a possible role?

**DOI:** 10.3389/fphar.2025.1538791

**Published:** 2025-05-12

**Authors:** Francesco Rettura, Christian Lambiase, Riccardo Tedeschi, Antonio Grosso, Lorenzo Cancelli, Angelo Ricchiuti, Andrea Bottari, Luca Giacomelli, Nicola de Bortoli, Massimo Bellini

**Affiliations:** ^1^ Gastrointestinal Unit, Department of Translational Research and New Technologies in Medicine and Surgery, University of Pisa, Pisa, Italy; ^2^ Regional Center for Functional and Motility Digestive Disorders, Department of Translational Research and New Technologies in Medicine and Surgery, University of Pisa, Pisa, Italy; ^3^ Gastroenterology Unit, Annunziata Hospital, Cosenza, Italy; ^4^ Polistudium SRL, Milan, Italy

**Keywords:** irritable bowel syndrome, IBS-D, gut barrier function, mucoprotectants, xyloglucan, gelatin tannate, intestinal permeability

## Abstract

Impaired gut barrier function plays a pivotal role in the pathophysiology of irritable bowel syndrome (IBS), particularly in IBS with diarrhea. Mucoprotectants, such as xyloglucan, gelatin tannate and pea protein tannins, offer a novel therapeutic approach by restoring intestinal permeability and reducing inflammation. This review assesses preclinical and clinical evidence supporting mucoprotectants in IBS with diarrhea management. Preclinical studies indicate their efficacy in reducing intestinal permeability and inflammation, while clinical trials demonstrate improvements in stool consistency, abdominal pain and bloating. Despite these promising results, comparative studies are needed to establish the superiority of specific mucoprotectants and their optimal use in clinical practice.

## 1 Introduction

Irritable bowel syndrome (IBS) is a chronic disorder of gut-brain interaction characterized by recurrent abdominal pain and altered bowel habits (constipation, diarrhea, or both) in the absence of a detectable organic cause (Rome IV criteria) (Drossman and Hasler, 2016; Mearin et al., 2016). This syndrome presents a worldwide prevalence ranging between 1.5% and 4.1% and is the most common functional gastrointestinal disorder encountered in primary and secondary care (Soncini et al., 2019; Bellini et al., 2022; Sperber et al., 2021). The pathophysiology of IBS is multifactorial and not fully understood (Mearin et al., 2016; Bellini et al., 2014). Impairment of intestinal permeability plays a pivotal role in IBS pathophysiology as it underlies visceral hypersensitivity, low-grade mucosal inflammation and changes in gut microbiota (Camilleri et al., 2012; Chey et al., 2015). Although IBS is not life-threatening, it profoundly impacts patients’ quality of life (QoL) and affects their psycho-affective profile (Portincasa et al., 2003). It is also associated with a significant socio-economic burden due to absenteeism from work, frequent diagnostic tests and medical checkups (Sandler et al., 2002; Buselli et al., 2021). Furthermore, the use of healthcare resources is increased by the lack of therapies that can comprehensively address IBS digestive symptoms and comorbidities (Bellini and Rossi, 2018; Ford et al., 2018).

The management of IBS with predominant diarrhea (IBS-D) remains a challenge for physicians; current therapeutic strategies aim to target individual symptoms. At present, there are several treatment options for IBS-D, including dietary approaches (e.g., Low-FODMAP diet – LFD), soluble fibers, psychological therapies, opioid agonists, mixed opioid agonists/antagonists (i.e., eluxadoline), loperamide, rifaximin, probiotics, 5-HT3 antagonists (e.g., ondansetron), antispasmodics agents, bile acid sequestrants (e.g., colestyramine), tricyclic antidepressant (TCAs) (e.g., amitriptyline) and selective serotonin reuptake inhibitors (SSRIs) (Barbara et al., 2023; Savarino et al., 2022). However, these therapeutic approaches often yield only partial and unsatisfactory results (Lucak et al., 2017), likely due to the complex and still poorly understood pathophysiology of IBS. Thus, major efforts are directed towards treating the predominant symptoms of IBS by targeting its underlying mechanisms (Ford et al., 2020). However, no medical therapy has been proven to modify the natural course of IBS or its fluctuating symptoms.

Given that an impairment of the intestinal barrier, which allows pathogen translocation and triggers an immune-inflammatory response, is a potential pathophysiological mechanism in IBS (Vicario et al., 2015; Fortea et al., 2021), an emerging therapeutic approach is aimed at restoring normal gut permeability. In this context, film-forming mucosal protective agents, known as mucoprotectants, offer a promising therapeutic alternative by enhancing and restoring gut barrier function (Eutamene et al., 2018). Recently, a variety of products combining different mucoprotectants (e.g., xyloglucan and gelatin tannate) have become available (Bellini et al., 2021; Inczefi et al., 2024). Although some evidence supports the efficacy of mucoprotectants in the treatment of both acute diarrhea and chronic inflammatory bowel disease (Gnessi et al., 2015; Pleşea Condratovici et al., 2016; Periasamy et al., 2018; Ross et al., 2021; Scaldaferri et al., 2014), there are still limited and fragmented data regarding their effectiveness in treating IBS patients.

After providing an overview of the structure and function of the intestinal barrier and its impairment in IBS, we review current knowledge on the role of mucoprotectants and propose some recommendations for their use in everyday clinical practice.

## 2 Intestinal barrier

**FIGURE 1:**
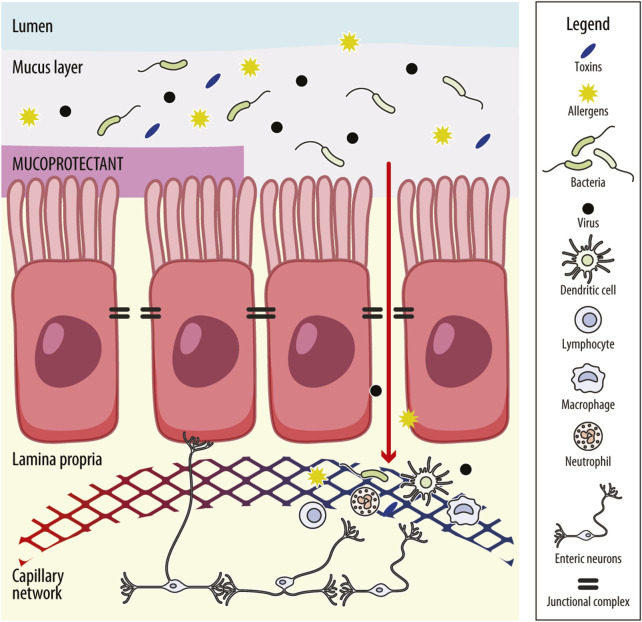
Schematic representation of the intestinal barrier and mechanism of action of mucoprotectants. The intestinal barrier comprises the mucus layer, the intestinal–epithelial barrier and the gut–vascular barrier. The mucus layer prevents the adhesion of pathogenic organisms to the epithelium. The intestinal–epithelial barrier consists of an enterocyte monolayer joined together by the junctional complex (i.e., tight junctions, adherens junctions, gap junctions and desmosomes) that maintains barrier integrity and regulates the paracellular trafficking of solutes and fluids. Molecules can cross the intestinal–epithelial monolayer also through the cells (transcellular route). The gut vascular barrier regulates the translocation of intestinal content into the systemic circulation and, in turn, into organs far from the intestine. It includes endothelial cells and enteric neurons placed in the lamina propria, where are also present innate immune cells (i.e., dendritic cells, macrophages and lymphoid cells). When the mucus layer is impaired, access by pathogens, toxins and allergens across the intestinal barrier is granted, which may enhance immune-inflammatory responses. This response, in turn, may lead to further distortion of intestinal permeability and perpetuation of mucosal low-grade inflammation, leading to visceral hypersensitivity. Mucoprotectants share mucoadhesive properties and the ability to create a film-forming barrier over the intestinal mucosa or protect the mucus layer, helping to restore gut permeability and avoid or decrease mucosal inflammation, reducing the effect of noxious agents on the intestinal barrier.

The intestinal barrier, which primarily consists of a mucus layer, an epithelial barrier and a gut–vascular barrier (GVB), plays a crucial role in health and disease by facilitating nutrient absorption and preventing the entry of pathogens (Figure 1; Pellegrini et al., 2023). The epithelium restricts access to noxious substances and secretes antimicrobial peptides, while the mucus prevents the adhesion of pathogenic organisms to the epithelium. Furthermore, the mucus layer provides a habitat for commensal gut bacteria, which helps to limit the colonization of pathogenic microorganisms. The intestinal epithelial barrier is formed by a monolayer of enterocytes interconnected through the junctional complex, which includes tight junctions, adherens junctions, gap junctions, and desmosomes. This complex is crucial for maintaining barrier integrity and regulating the paracellular transport of solutes and fluids. Among these structures, tight junctions are the most apical and consist of multi-protein assemblies composed of transmembrane proteins (such as claudins and occludins), peripheral membrane proteins (including zonula occludens [ZO]), and regulatory molecules such as kinases (Turner, 2009). Increased intestinal permeability has been reported in 37%–62% of patients with IBS-D (Hanning et al., 2021; Dunlop et al., 2006), though it is also present in other subgroups of IBS. Impaired intestinal permeability, particularly in IBS-D, is associated with a structural reduction in the expression of tight junction proteins, such as occludins, ZO-1 and claudins, compared to healthy individual (Dunlop et al., 2006). Genetic predisposition, stress, adverse food reactions, bile acid malabsorption and the excessive release of proteolytic mediators may contribute to permeability alterations (Camilleri, 2022; Barbara et al., 2021; Sciumè et al., 2023). Diet also modifies intestinal permeability, including fat and emulsifiers that increase permeability, and nutrients, such as fibre, glutamine, zinc, vitamin D, polyphenols and anthocyanins, that decrease permeability (Matar et al., 2024).

Impaired intestinal permeability plays a significant role in the development of IBS symptoms (Bellini et al., 2021). Increased gut permeability poses a challenge to the mucosa, exposing it to luminal antigens, microbiota and their metabolites, which promotes and sustains mucosal immune activation and visceral hypersensitivity. Notably, increased intestinal permeability in IBS correlates with the severity of abdominal pain (Piche et al., 2009). Conversely, restoring barrier function improves both abdominal pain and visceral hypersensitivity (Barbara et al., 2016; Zhou et al., 2019; Long et al., 2018).

The role of the gut barrier in the pathogenesis of gastrointestinal disorders has been explored, focusing on both epithelial and vascular permeability. Interestingly, markers of epithelial permeability were found to be more strongly associated with abdominal symptoms, whereas markers of vascular permeability correlated more closely with psychological symptoms (Carloni et al., 2021; Barbaro et al., 2024). A study conducted in a murine model of dextran sodium sulfate-induced colitis suggested that impairment of the GVB, with a subsequent increase in the endothelial cell-specific plasmalemma vesicle-associated protein (PV1), a marker of vascular permeability, is linked to psychological symptoms (Carloni et al., 2021). Another study, conducted both on colonic biopsies from IBS patients and *in vitro* using an intestinal–epithelial barrier model with the human intestinal epithelial cell line Caco-2, showed that epithelial barrier integrity is compromised throughout the entire gastrointestinal tract, particularly in IBS-D patients (Barbaro et al., 2024). Therefore, identifying specific agents that prevent intestinal barrier dysfunction and reduce intestinal permeability could represent a novel therapeutic approach to treating IBS (Camilleri et al., 2012).

## 3 Mucoprotectants: mechanisms of action

Mucoprotectants are compounds of various types (e.g., insoluble salts, hemicellulose, tannic acid, gelatins) with the ability to enhance the intestinal barrier by forming a film over the intestinal mucosa, thereby reducing the impact of pathogens and improving the function of the intestinal barrier (Alonso-Cotoner et al., 2021). These compounds act intraluminally to modify enteric contents and may serve as an alternative or complementary therapy for managing acute and chronic diarrheal disorders (Aloi and Mennini, 2019). Several mucoprotectant products, classified as class IIa or III medical devices, have been approved in European countries for the restoration of intestinal wall function and the treatment of diarrhea (Eutamene et al., 2018; Lopetuso et al., 2015). Table 1 outlines general information on mucoprotectants and their mechanisms of action.

**TABLE 1:** General information on mucoprotectants and their mechanisms of action.

Mucoprotectant	Components	Mechanism of action	Additional information
Gelatin tannate	Combination of gelatin and tannic acid (penta-m- digallolyl-glucose)	Forms a stable, non-dissociated mucoadhesive film in the intestine, providing mechanical protection and influencing microbiota composition	Remains stable through the stomach
Xyloglucan	Polysaccharide hemicellulose from *Tamarindus indica* (tamarind tree) seeds	Forms a protective film over the intestinal mucosa, protecting against pathogens and improving barrier function	Not broken down by digestive enzymes
Pea protein and tannins	Pea protein and tannins from grape seed extract	Exhibits strong antioxidant activity by inhibiting lipid peroxidation, lipoxygenases and scavenging radicals	*In vitro* activity includes inhibition of lipid peroxidation

### 3.1 Gelatin tannate

Gelatin tannate (GT), a stable combination of gelatin and tannic acid (TA; penta-m-digallolyl-glucose), passes unaltered through the stomach. Upon reaching the intestine, it acts in its non-dissociated form as a mucoadhesive film (Lopetuso et al., 2017), which is formed through electrostatic bonds between gelatin tannate and mucins (Freli et al., 2013). It was previously thought that GT was hydrolyzed into gelatin and TA in the intestine, with TA responsible for its mucoprotective and antidiarrheal effects (Frasca et al., 2012; De Servi et al., 2012). In their review, Ruszczyński et al. extensively explained TA’s activity and suggested that TA may be responsible for both GT’s mechanism of action and its potential adverse effects (Ruszczyński et al., 2014). GT remains a stable complex within the intestine and, in its undissociated form, provides mechanical protection to the mucosa (Bueno et al., 2013). It may also influence the composition of the intestinal microbiota (Scaldaferri et al., 2014; De Servi et al., 2012).

### 3.2 Xyloglucan

Xyloglucan (XG) is a water-soluble polysaccharide hemicellulose extracted from the seeds of the tamarind tree (*Tamarindus indica*) and is not broken down by digestive enzymes. It forms a film over the intestinal mucosa, helping to protect against pathogens and improve intestinal barrier function. XG is often combined with gelatin to prolong its availability within the intestine (Gnessi et al., 2015; Bueno et al., 2014). XG also exhibits protective effects against bacterial invasion and alterations in intestinal permeability (Eutamene et al., 2018; Piqué et al., 2018).

### 3.3 Pea protein and tannins

Pea protein and tannins (PPT) from grape seed extract are also mucoprotective agents (Trifan et al., 2019). PPT complexes demonstrate strong antioxidant activity by inhibiting *in vitro* lipid peroxidation, lipoxygenases and scavenging free radicals (Serrano et al., 2009).

## 4 Mucoprotectants: preclinical studies

Many *in vitro* and *in vivo* studies have been conducted on XG, GT and PPT, both alone and in combination (Table 2).

**TABLE 2:** Summary of preclinical studies on mucoprotectants.

Study	Studied drugs	Model	Results
*In vitro* studies			
Frasca et al. (2012)	GT	Caco-2 cells exposed to LPS	GT reduced IL-8 and TNF-α expression in a dose-dependent manner
Bueno et al. (2014)	XG	Cell cultures exposed to *E. coli*	XG improved trans-epithelial electrical resistance and reduced *E. coli* colonization when used preventively
De Servi et al. (2012)	XG + Gelose	Caco-2 and CacoGoblet™ cells inoculated with *E. coli*	XG + Gelose preserved tight junctions, reduced intercellular permeability and prevented *E. coli* invasion
Fraile et al. (2017)	XG, propolis and hibiscus	CacoGoblet™ and RWPE- 1 cells exposed to uropathogenic *E. coli* strains	The combination avoided bacterial contact with cell monolayers and acted as a bioprotective barrier without affecting *E. coli* cell integrity
Campolo et al. (2020)	XG + PP	HaCaT keratinocytes exposed to *S. aureus* infection	XG + PP improved membrane integrity and reduced bacterial adherence
*In vivo* studies			
Bueno et al. (2013)	GT + TA	Rats with *E. coli* LPS- induced enteritis	GT reduced jejunal tight junction permeability by 78.1% and decreased MPO activity. TA and gelatin alone had no effect
Bueno et al. (2014)	XG	Rats with *E. coli* LPS- induced enteritis	XG reduced mucosal permeability and mitigated cholera toxin-induced secretory effects
Esposito et al. (2018)	XG + Gelose	Sprague-Dawley rats infected with *S. enterica* or *E. hirae*	XG + Gelose reduced inflammation and increased occludin and ZO-1 levels without bactericidal effects
Filippone et al. (2022)	XG + PP + Chia Seed	Sprague-Dawley rats (Control, IBS-C model, IBS-C model + XG + PP + Chia Seed)	Increased stool moisture, improved mucosal alterations and increased occludin and ZO-1 expression
Scuderi et al. (2022)	XG + PP	Sprague-Dawley rats with PRS and CRD	XG + PP prevented visceral hypersensitivity, reduced intestinal permeability and lowered IL-1β and IL- 6 levels
Eutamene et al. (2022)	GT + XG	Rat models of CT-induced water secretion	GT and XG attenuated CT-induced water secretion in rats, supporting mucoprotectant mucoadhesive film formation
Theodorou et al. (2023)	XG + Gelatin or Gelose	Rats with *E. coli* LPS- induced enteritis	XG combined with gelatin (250 mg/kg) or gelose (250 or 500 mg/kg) reduced LPS-induced jejunal hyperpermeability and inflammation
Inczefi et al. (2024)	XG + PP + PPGS + XOS	Wistar rats with PRS	7-day treatment with XG + PPGS + XOS reversed PRS-induced rectal hypersensitivity and gut hyperpermeability

CRD: colorectal distension; CT: cholera toxin; GT: gelatin tannate; IBS-C: irritable bowel syndrome with constipation; LPS: lipopolysaccharide; MPO: myeloperoxidase; PP: pea protein; PPGS: grape seed extract; PRS: partial restraint stress; TA: tannic acid; XG: xyloglucan; XOS: Xylo-oligosaccharides; ZO-1: Zonula Occludens-1.

### 4.1 *In vitro* studies

In an intestinal mucosa model composed of Caco-2 and CacoGoblet™ cells, a mixture of XG and gelatin was shown to effectively preserve tight junctions, thus reducing intercellular permeability and preventing *Escherichia coli* invasion by creating a protective physical barrier (de Servi et al., 2016). GT also demonstrated anti-inflammatory effects by inhibiting the release of TNF-α and IL-8 and reducing ICAM-1 expression in the lipopolysaccharide (LPS)-exposed intestinal model (Frasca et al., 2012). In cell cultures exposed to *E. coli*, XG improved trans-epithelial electrical resistance, an indicator of tight junction permeability, and, when used preventively, reduced *E. coli* colonization (Bueno et al., 2014). Similarly, the different combinations of XG with gelatin or PPT produced protective results in other epithelial cell models, such as those of the urinary tract (Fraile et al., 2017) and skin (Campolo et al., 2020), respectively.

### 4.2 Animal models

A study found that XG combined with gelatin (250 mg/kg) or gelose (250 or 500 mg/kg) had beneficial and comparable effects on intestinal permeability and inflammation in a rat model of *E. coli* LPS-induced enteritis (Theodorou et al., 2023). Eutamene et al. evaluated the mucoprotective effects of GT, XG and related compounds in rat models of cholera toxin (CT)-induced water secretion. The mucoprotectants attenuated CT-induced intra-loop water secretion, supporting earlier evidence that their mucosal protection mechanisms are closely related to their chemical structures, which confer film-forming properties via mucoadhesive films (Eutamene et al., 2022).

In a model of enteritis induced by *E. coli* lipopolysaccharides, GT reduced jejunal tight junction permeability, whereas this effect was not observed with TA or gelatin alone. Six hours after LPS injection, both jejunal tight junction permeability and MPO activity increased significantly in rats. Oral pretreatment with GT reduced the jejunal permeability increase by 78.1%, while gelatin and TA had no effect. These findings suggest that only the stable complex of gelatin and TA has the potential to form a biofilm and provide GT’s protective effects (Bueno et al., 2013). In another *in vivo* animal model, XG reduced the increase in mucosal permeability caused by the intraperitoneal injection of *E. coli* lipopolysaccharides and reduced the secretory effects induced by cholera toxin (Bueno et al., 2014).

A 2022 study by Filippone et al. (2022) compared three groups of rats: a control group, an IBS-C-induced model and an IBS-C model that received a combination of XG, pea protein and chia seed powder for 7 days. The study demonstrated a reduction in constipation, with increased food and water intake, reduced weight loss and improved cytoarchitectural damage, as well as increased expression of Occludin and ZO1 in the group treated with the XG, PP and CS combination. Similarly, Scuderi et al. (2022) showed a reduction in visceral hypersensitivity, abdominal distension and intestinal permeability in a rat model exposed to partial restraint stress and colorectal distension that also received treatment with XG and PP. In a recent study by Inczefi et al. (2024), Gelsectan^®^ (a combination of xyloglucan, pea protein and other compounds) was tested in rats exposed to partial restraint stress (PRS). The study showed that a 7-day oral administration of Gelsectan^®^ reversed PRS-induced rectal hypersensitivity and gut hyperpermeability, suggesting its efficacy in restoring gut barrier function.

The combination of XG and gelose in animal models of gastroenteric and urinary tract infections caused by *Salmonella enterica* and *Enterococcus hirae* significantly reduced intestinal permeability, neutrophil infiltration of the mucosa and overall histological damage (Esposito et al., 2018). These findings suggest that XG and gelose play a protective role by coating the intestinal mucosa with a protective layer. The protective effects of the XG and gelose combination on urinary tract infections were also confirmed in another study by the same author (Esposito et al., 2020).

## 5 Mucoprotectants: clinical efficacy

Many clinical studies have been conducted on XG, TA GT and PPT, both alone and in combination (Table 3).

**TABLE 3:** Summary of clinical studies on mucoprotectants.

Study	Studied drugs	Study design	Population	Results
Durbán Reguera et al. (2007)	GT + ORS	Community-based multicenter observational study	54 adults with acute diarrhea	Significant improvement in bowel movements and stool consistency within 12 h of treatment
Esteban et al. (2009)	GT + ORS	Comparative study	Pediatric patients (<3 years) with acute diarrhea	GT + ORS led to a faster reduction in stool number and improvement in stool consistency
Allegrini and Costantini (2012)	GT	Double-blind RCT	40 adults with acute diarrhea	GT reduced stool frequency and abdominal pain more effectively than placebo. Safe and well-tolerated
Gnessi et al. (2015)	XG	Multicenter RCT	150 adults with acute diarrhea	Faster relief from symptoms, including stool consistency and abdominal discomfort
Pleşea Condratovici et al. (2016)	XG + ORS	Multicenter RCT	36 children with acute gastroenteritis	XG + ORS resulted in faster symptom improvement compared to ORS alone. Effective and safe
Alexea et al. (2016)	Oligo/Polysa ccharides, GT, Gelose	Double-blind RCT	128 IBS-D patients	Reduced abdominal pain and flatulence, with improved quality of life
Russo et al. (2018)	GT + Flavonoids	Case-controlled trial	60 children with acute diarrhea	Stool frequency was reduced, though diarrhea duration was similar between groups
Trifan et al. (2019)	XG + PPT + XOS	Double-blind crossover trial	60 IBS-D patients	Stool normalization and symptoms improved with XG + PPT + XOS compared to placebo
de Los Rios et al. (2021)	XG + PPT + XOS	Multicenter observational study	50 IBS-D patients	Improvement in IBS symptoms, including diarrhea and pain, over time
Santos et al. (2021)	XG + Gelose	Double-blind RCT	100 children with acute diarrhea	XG + gelose combined with ORS improved stool consistency and reduced diarrhea

GT: gelatin tannate; ORS: oral rehydration solution; PPT: pea protein tannins; RCT: randomized controlled trial; XG: xyloglucan; XOS: xylo-oligosaccharides.

### 5.1 Gelatin tannate

A double-blind, randomized, placebo-controlled trial conducted in an Italian general practice setting involved 40 adults with acute diarrhea due to intestinal infection. Participants were treated with GT 500 mg (n = 20) or placebo (n = 20) six-times daily for 2 days. A significantly greater reduction in the frequency of watery stools (assessed using the Stool Decrease Index) and in the severity of abdominal pain (assessed using a visual analog scale) was observed in the GT group compared with the placebo group (both p < 0.01). Significantly more patients in the GT group than in the placebo group were classified as responders, defined as a reduction of at least 30% in both stool and pain indices (85% versus 25%, p < 0.001). GT was safe and well tolerated, with no adverse events or changes in laboratory parameters reported (Allegrini and Costantini, 2012).

A community-based, multicenter, prospective observational study in Spain evaluated the effect of GT plus oral rehydration in 54 adults with acute diarrhea (reported in a poster). Diarrhea improved significantly 12 h after starting treatment: the number of patients experiencing more than four bowel movements per day decreased from 85.2% at baseline to 0% at 12 h. Stool consistency was watery in all patients at baseline, but at 12 h, 39.1% had soft stools and 60.9% had normal stools. Bloody diarrhea, present in 15.4% of patients at baseline, was absent at 12 h. The incidence of vomiting also decreased and the body temperature returned to normal (Durbán Reguera et al., 2007).

There is also some evidence from studies in pediatric populations. One study compared the response at 12 h from baseline between two cohorts of pediatric patients (less than 3 years old) with acute diarrhea treated with oral rehydration solution (ORS) alone or ORS plus GT. A significant decrease in the number of stools and an improvement in stool consistency were observed in the ORS + GT group (Allegrini and Costantini, 2012). In a meta-analysis of three RCTs on the use of GT combined or not with ORS, GT was demonstrated to improve stool frequency and consistency in children with acute gastroenteritis (Aloi and Mennini, 2019).

One of the first trials using oligosaccharides, polysaccharides and reticulated protein (a mixture of tannins and gelose) on IBS-D patients (diagnosed following Rome III criteria) dates to 2016 (Alexea et al., 2016). Its results display a significant improvement in abdominal pain and flatulence in patients treated with the oligo/polysaccharide compound compared with those receiving placebo.

### 5.2 Xyloglucan in acute diarrhea

A multicenter randomized controlled trial (RCT) in children (aged 3 months to 12 years) with acute gastroenteritis of infectious origin evaluated the efficacy, safety and onset of the antidiarrheal effect of XG. Children were randomized to receive either XG plus ORS or ORS alone for 5 days. Patients receiving XG and ORS experienced better symptom evolution than those who received ORS alone, with a faster onset of action. At 6 h, XG produced a significantly greater decrease in the number of type 7 stools (p = 0.027). On days 3 and 5, XG also resulted in a significantly greater reduction in types 6 and 7 stools compared with ORS alone. XG was effective and safe for treating acute gastroenteritis in children and had a rapid onset of action in reducing diarrheal symptoms (Pleşea Condratovici et al., 2016).

The efficacy of XG in treating acute diarrhea was also compared to diosmectite and *S. Boulardii* in a randomized, multicenter, open-label study involving 150 patients. Patients were randomly assigned to receive one of the three treatments. XG showed a faster onset of action and greater improvement in diarrheal symptoms during the first 24 h of treatment, although statistical comparisons were not reported. All three treatments were well tolerated and no adverse events occurred during the study. XG was also more effective in reducing nausea, vomiting, abdominal pain and flatulence (Gnessi et al., 2015).

In 2020, Santos et al. (2021) conducted a randomized, double-blind, placebo-controlled trial on the efficacy and safety of XG plus gelose in combination with ORS for treating acute diarrhea in children. The study found that XG plus gelose and ORS significantly reduced the number of liquid and mushy stools compared to ORS alone. It also showed positive effects in reducing additional symptoms, such as vomiting, apathy and flatulence.

Similar results were achieved using Actitan-F (a complex containing tannates and flavonoids) added to ORS in children with acute diarrhea. Actitan-F reduced the mean number of stools compared to baseline, although it did not shorten the total duration of symptoms (Russo et al., 2018).

### 5.3 Protein and tannins

Some studies have focused on evaluating the efficacy of combinations of mucoprotectants, often in conjunction with xylo-oligosaccharides (XOS), which have antioxidant activity by reducing reactive oxygen species and exert a prebiotic effect by increasing Bifidobacteria in the gut microbiota (Huang et al., 2019; Finegold et al., 2014). In a double-blind, crossover trial, Trifan et al. (2019) found that a combination of XG, PPT and XOS effectively controlled abdominal pain and bloating, reduced bowel movements and improved perceived quality of life after 28 days of treatment in IBS-D patients.

A 2021 multicenter prospective observational study on 50 IBS-D patients treated twice daily with XG, PPT and XOS for 6 months reported an improvement in symptoms (based on the IBS-SSS questionnaire) and bowel habits. The treatment had an excellent safety profile, with few adverse effects, which were mild and unrelated to the treatment, even with long-term use (de Los Rios et al., 2021).

## 6 Discussion

The complexity of IBS pathophysiology, particularly in IBS-D, underscores the importance of addressing intestinal permeability and mucosal integrity as key therapeutic targets. Mucoprotectants offer a promising approach due to their ability to restore and enhance the gut barrier without systemic absorption. Thus, it indirectly reduces the exposure of the submucosal neuronal and immune systems to luminal triggers. This unique mechanism addresses a critical gap in IBS-D management, where symptom control remains challenging and patient satisfaction with existing therapies is low.

Overall, mucoprotectants, such as XG, GT and PPT, show promising preclinical and clinical efficacy. Preclinical studies highlight that mucoprotectants reduce intestinal permeability, prevent bacterial invasion and mitigate inflammatory responses in animal models of enteritis and gut dysfunction. For instance, XG combined with gelose and GT demonstrated significant effects in reducing intestinal permeability and protecting tight junction integrity in rat models of *E. coli*-induced enteritis.

Clinical studies further support these findings. Clinical studies in both adult and pediatric populations show that XG and GT can reduce diarrheal symptoms and improve stool consistency. In IBS-D patients, a combination of XG, PPT and XOS significantly reduced abdominal pain, bloating and bowel movement frequency. In line with these positive outcomes, European guidelines indicate mucoprotectants as a promising therapeutic approach for patients with IBS-D (Savarino et al., 2022). However, the overall evidence remains limited, with studies often underpowered and lacking long-term follow-up.

There is currently no clear indication that any specific mucoprotectant is superior, either alone or in combination. Comparative studies would be highly valuable to determine the relative efficacy of these agents. However, the combination of XG, PPT and XOS appears to have the strongest evidence of efficacy (Bellini et al., 2021; Inczefi et al., 2024; Trifan et al., 2019; Lucca et al., 2024). In clinical settings, XG-based therapies have shown benefits in improving symptoms of acute diarrhea and IBS-D, including reductions in stool frequency, abdominal pain and bloating. However, more robust, head-to-head trials are needed to confirm whether the combination of XG, PPT and XOS provides superior outcomes compared to other mucoprotectants.

From a practical perspective, mucoprotectants present a viable first-line treatment for IBS-D, particularly in patients with mild to moderate symptoms, due to their excellent safety profile and low incidence of adverse effects, making them suitable for long-term use. While clinical observations have not reported significant effects on the absorption of other medications or nutrients, this has not been conclusively demonstrated through experimental studies. Therefore, although mucoprotectants are commonly used in combination with other IBS-D therapies without apparent reductions in efficacy, further research is needed to confirm their impact on drug and nutrient absorption. In clinical practice, dietary modifications and probiotics remain widely used treatment options for IBS-D (Soncini et al., 2019). While current guidelines offer a weak recommendation for probiotics in IBS management due to limited supporting clinical evidence (Ford et al., 2018; Barbara et al., 2023; Savarino et al., 2022), combining probiotics with mucoprotectants may yield complementary effects on IBS-D pathophysiology. Specifically, mucoprotectants can reduce impaired intestinal permeability, whereas probiotics can target dysbiosis, a factor commonly associated with gut barrier dysfunction (Camilleri, 2019), potentially enhancing overall clinical outcomes. Additionally, mucoprotectants could bolster the effects of the low-FODMAP diet (LFD), a dietary strategy increasingly applied in IBS management (van Lanen et al., 2021; Bellini et al., 2020a; Rettura et al., 2023; Lambiase et al., 2024). Mucoprotectants may improve the tolerability of a strict LFD in the mid-term as patients identify FODMAP triggers, or even enable an adapted LFD at an earlier phase, thereby providing significant benefits for patients in terms of cost reduction, reduced risk of nutritional deficiencies, and improved adherence (Bellini et al., 2020b).

The potential for combining multiple mucoprotectants or integrating them into broader IBS management strategies warrants further investigation. However, significant gaps remain in our understanding, particularly regarding their role in modulating the gut microbiota and addressing dysbiosis in IBS-D. Future studies should evaluate whether specific mucoprotectant combinations offer synergistic benefits in improving both intestinal permeability and microbiota balance. Additionally, key areas requiring further research include their mechanisms of action, pharmacokinetics, and potential interactions with other therapies. Current clinical evidence is limited by small sample sizes, short follow-up durations, and methodological inconsistencies. Well-designed, large-scale randomized controlled trials and mechanistic studies are essential to confirm their efficacy, safety, and role in gut barrier function and immune modulation, ultimately supporting their inclusion in clinical guidelines.

In conclusion, while mucoprotectants hold promises for treating IBS-D by targeting gut barrier dysfunction, more robust data are required to validate their role and determine the most effective therapeutic combinations.
